# Immediate effects of muscle energy technique and stabilization exercise in patients with chronic low back pain with suspected facet joint origin: A pilot study

**DOI:** 10.1142/S1013702520500109

**Published:** 2020-03-20

**Authors:** Wahyuddin Wahyuddin, Mantana Vongsirinavarat, Keerin Mekhora, Sunee Bovonsunthonchai, Rachaneewan Adisaipoapun

**Affiliations:** 1 Faculty of Physical Therapy, Mahidol University, Nakhon Pathom, Thailand; mantana.von@mahidol.edu

**Keywords:** Chronic low back pain, facet joint, lumbar stabilization, muscle energy technique

## Abstract

**Background:**

Facet joint is a potential structure to be the source of chronic low back pain (LBP) affecting lumbar motion, pain, and disability. Other than the recommended treatment of lumbar stabilization exercise (LSE), several manual procedures including muscle energy technique (MET) are commonly used in physical therapy clinic. However, little evidences of the effects of MET have been reported.

**Objective:**

This study aimed to compare the immediate effects of MET and LSE in patients with chronic LBP with suspected facet joint origin.

**Methods:**

Twenty-one patients with low back pain were recruited and randomly assigned to receive treatment either MET or LSE. The outcomes were kinematic changes, pain intensity, and disability level. Lumbar active range of motion (ROM) of flexion, extension, left and right lateral flexion, and left and right rotation were evaluated using the three-dimension motion analysis system at baseline and immediately after treatment. Pain intensity was evaluated using visual analogue scale (VAS) at baseline, immediately after, and two days after treatment. Thai version of the modified Oswestry disability questionnaire (ODQ) was utilized at baseline and two days after treatment. The mixed model analysis of variance was used to analyze all outcomes.

**Results:**

The results showed that all outcomes were not different between groups after treatments. Although there were statistically significant improvements after the treatments when collapsing the groups, the minimal clinically important change was found only for pain but not for lumbar movements and disabilities scores.

**Conclusion:**

The effect of MET and LSE alone in single session might not be intensive enough to improve movements and decrease disability in patients with chronic LBP with suspected facet joint origin.

## Introduction

Facet joint has been implicated as the cause of chronic pain in the lower back due to the possible pathoanatomical mechanism.^1^ The prevalence of facet joint pain was estimated as high as 75% among people reporting low back pain (LBP).^2^ In a community-based survey, the prevalence of lumbar facet osteoarthritis reportedly increased with age i.e., 89.2% in persons age more than 60 years, although the association between LBP and osteoarthritis identified by computed tomography was not apparent.^3^ The assumed characteristics of acute facet joint pain include local, unilateral, decreased motions in extension and rotation, occasionally pain extending to thigh, no neurologic signs, and aggravation of pain in flexion, sitting, coughing or sneezing, and no antalgic posture.^4^ The clinical indicators of LBP with facet joint origin have been consensus by an expert panel and suggested to make the patients more homogeneous and appropriate for investigating effect of specific interventions.^5^

Decreased lumbar motions, as well as an increased pain and disability are the main impairments in patients with chronic LBP including ones with facet joint origin. The possible mechanism is the forces on articular facets which could stretch the joint capsules and the sinu-vertebral capsular nerve might be irritated and provoking muscular guarding.^6^ Joint inflammation, degeneration and trauma are then associated with pain during movement, and lead to movement and functional limitation.^7^ A variety of manual and exercise techniques are used in clinics to solve these complaints with little evidences on movement improvement. A study was conducted previously to test the effect on the kinematic of osteopathic manipulative treatment combined with specific exercise in patients with chronic LBP.^8^ However, the study only measured the forward flexion in sagittal plane.

The Muscle Energy Technique (MET) has been suggested for treating patients with LBP especially ones with facet joint dysfunction.^9^ However, the reports of the MET effectiveness were mostly based on asymptomatic subjects^10,11^ or cases with heterogeneous clinical pictures.^12^ Studies using MET showed its effectiveness to improve disability and functional level in patients with acute LBP^13^ and a short-term positive effect in patients with lumbopelvic pain.^14^ For chronic cases, the evidences of MET effectiveness are still very limited. A study of MET compared with Maitland’s mobilization presented moderately better outcomes of function and range of motion (ROM) in the subjects with chronic LBP due to sacroiliac joint dysfunction.^15^ The six-day intervention of MET compared with conventional physical therapy also showed that the MET could restore functional leg length difference to nearly normal. However, both groups presented similar results for pain and disability.^16^

From a physiological perspective, MET serves the treatment goals of patients with chronic LBP with facet joint origin in restoring motion and eliminating muscle spasm.^6^ MET procedures focus on identifying the restriction and mobilizing joints and tissues through the local muscle effort.^17^ The proposed treatment mechanisms involve the neurological and biomechanical responses, including hypoalgesia, proprioception, motor control, and changes of tissue fluid.^18,19,20^ The pain alleviated mechanisms involve central and peripheral modulations such as activation of muscle and joint mechanoreceptors and centrally neural mediated pathways.^21^ Localized mobilization using MET might also inhibit the motor neuron activity which effectively relaxes the motion segment as well as normalizes the proprioceptive and motor coordination of the involved region.^22,23^

The lumbar spinal stabilization exercise is a common intervention used in physical therapy clinic for treating LBP patients.^24^ A systematic review supported the evidence that lumbar stabilization exercise (LSE) could reduce pain and disability level in patients with chronic LBP.^25^ The main focus of this treatment is also to improve the neuromuscular control of lumbar spine by the trunk muscles,^24,25^ therefore it was chosen to be the control intervention in this study.

The evidence to support the results of MET for symptomatic subjects was limited. Therefore, this study aimed to compare the immediate effects of MET and LSE for patients with chronic LBP with clinical symptoms of suspected facet joint origin on active ROM, pain intensity and disability level.

## Methods

This pilot study was compared between two groups of LBP patients who were treated with two different treatments i.e., the MET and the LSE. Randomized group allocation and blinded assessor procedure were applied. The outcomes were the kinematic changes, pain intensity, and disability level. This study was conducted after the protocol was approved by the Ethics Committee Mahidol University Institutional Review Board (MU-IRB, COA. No. 2014. 033.2103, Protocol No. MU-IRB 2014/006.0901). This study was registered with the ISRCTN, trial registration code ISRCTN 18528219.

The subjects were the patients with chronic LBP with the clinical symptoms of facet joint origin from the Physical Therapy Center, Mahidol University. All subjects who participated in this study signed the informed consent before commencing the study. The inclusion criteria were the age range of 18–60 years, recurrent or chronic LBP for at least three months, and pain severity from mild to moderate (21–69 mm on Visual Analogue Scale (VAS)). Nine out of 11 diagnostic criteria of facet joint pain origin according to Wilde *et al.* were used since they were applicable and routinely used in physical therapy clinic.^5^ The criteria consisted of (1) localized unilateral back pain, (2) referred pain not exceeding the knee level, (3) no sign of a nerve root irritation (dermatomal pain and paraesthesia) and nerve root compression (dermatomal sensory loss, myotomal weakness, and loss of reflex), (4) pain aggravated by pressure over lumbar facet joint, (5) pain aggravated in extension, (6) pain aggravated in three plane movements (extension, lateral flexion, and rotation) to the ipsilateral side, (7) pain eased in flexion, (8) increased stiffness of the facet joint during passive accessory movement, and (9) unilateral muscle spasm over the affected lumbar facet joint. The interrater reliability of this examination scheme in patients with LBP was reported to be acceptable (86.7%), with percent agreements for items ranging from 73.3% to 91.1%, and overall Kappa coefficient of 0.492 (p=0.001).^26^

The three dimension motion analysis system (Vicon Motion Systems Ltd., Oxford, UK) was used to assess the lumbar ROM. The reflective markers were attached on the 12th thoracic segment, the left and right anterior superior iliac spines, and the midpoint between the left and right posterior superior iliac spines.^8,27^ To prevent random errors from raw data that might contain additive noises from many sources during data collection, harmonic or frequency analysis was conducted. Residual analysis to choose cut-off frequency and filter technique with Butterworth was used. The cut-off frequency in this study was 5 hertz. The movements measured included flexion, extension, right and left lateral flexion and rotation. All movements were repeated three times, and the average value was used in the analyses. The measurement errors of this system were 7.39°, 6.75°, 2.25°, and 2.05° for flexion, extension, lateral flexion, and rotation, respectively.^28^

To assess pain intensity, the horizontal 100 mm VAS with two ends labeled as ‘no pain’ and ‘pain as bad as it could be’ was used.^29^ The subjects were asked to put a mark on the line to represent their level of pain intensity. The Thai version of modified Oswestry Disability Questionnaire (ODQ) was applied to assess the level of disability related to LBP. The test–retest reliability of this questionnaire was reportedly excellent (ICC=0.98).^30^ The assessor was a therapist who was blinded to the information of the group allocation. After screening the patients to confirm the eligibility, the assessor performed all pre-treatment measurements. The subjects were then asked to move to the separated treatment area.

To assign the group allocation, another staff drew a label of group from an opaque box. The treatments according to the predetermined protocol for each group were then applied. The treatments for both groups were provided by the same physical therapist with 21 years of experience in the musculoskeletal field. After treatments, the patients went back to the evaluation section and all lumbar active ROMs were re-evaluated immediately after the treatments by the same assessors. The pain intensity was recorded immediately post-treatments and on the next visit which for our research setting was normally two days after the first treatment. The disability level measurement was also conducted during the following visit.

For the MET group, the selection of technique was based on the symptoms and diagnosis of the direction of the dysfunction according to the textbook “Greenman’s principles of manual medicine”.^31^ The dysfunction diagnostic procedure included the assessment of the paired transverse processes in neutral, extended and flexed positions. Started with spinous processes palpation to mark the lumbar vertebra level, the transverse processes of both sides were then identified. The levels of transverse processes were first assessed in the neutral, and extended with prone position on the bed. The fully flexed position was also assessed with the patient seated on a stool with the feet on the floor. If transverse process of one side was more posterior in the flexed position and symmetric in the extended position, the restriction with extension, rotation, and side bend (ERS) dysfunction was posited. If one transverse process was more prominent in the extended position and symmetric in the fully flexed position, the restriction with flexion, rotation, and side bend (FRS) was posited. A neutral dysfunction was suspected if three or more transverse processes were prominent in all positions. The direction for treatment was the same with movement barrier and opposite with positional diagnosis. Prior to the treatment, the therapist arranged the appropriate position and assessed the pain or resistance barrier of the subject. The physical therapist used palpation to monitor the dysfunction segment and muscle contraction at that specific level during treatment.^31^ Since the pain was chronic, a light to moderate active contraction force was applied. After the contraction effort, the subject was instructed to completely relax the back, and the therapist reengaged movement limitation. Before repositioning to a new barrier resistance, the subject was relaxed and his/her muscles could be stretched to a new resting length. These procedures were repeated for about three to five times.^9^

For the LSE group, the procedure of exercise to relearn a precise co-contraction pattern of the deep trunk muscles including the transversus abdominis and lumbar multifidus muscles was conducted.^32^ Before the exercise, the abdominal breathing and abdominal hollowing were practiced as training. The exercises were performed in supine position including abdominal hollowing, unilateral knee abduction, extension and knee raise, and bilateral knee raise. The levels of exercise and progressions were customized for each subject, depending on the ability to learn to perform the co-contracting of the lumbar stabilizers. Approximately, 25 min was taken to complete the session for both MET and LSE treatments.

Since there was no previous study on the immediate effect of MET on the kinematic data, the sample size calculation was based on the most resembling of specific exercise and osteopathic manipulative therapy (OMT).^8^ The authors compared the kinematic data, pain, and disability from two groups of treatments: the group which was treated with specific exercises (SE) plus OMT, and the other group with the SE alone. The calculation with criteria of 80% power and α=0.05 indicated that 18 subjects of each group was required.^8^

To analyze the data, all results gained in this study were computed with the IBM SPSS Statistics for Windows, Version 19.0 (Armonk, NY: IBM Corp). Besides, the mixed model analysis of variance was applied for the analysis of all outcomes. Also, the independent sample t-test was used to compare the personal characteristics between two groups of subjects.

## Results

**Fig. 1.**
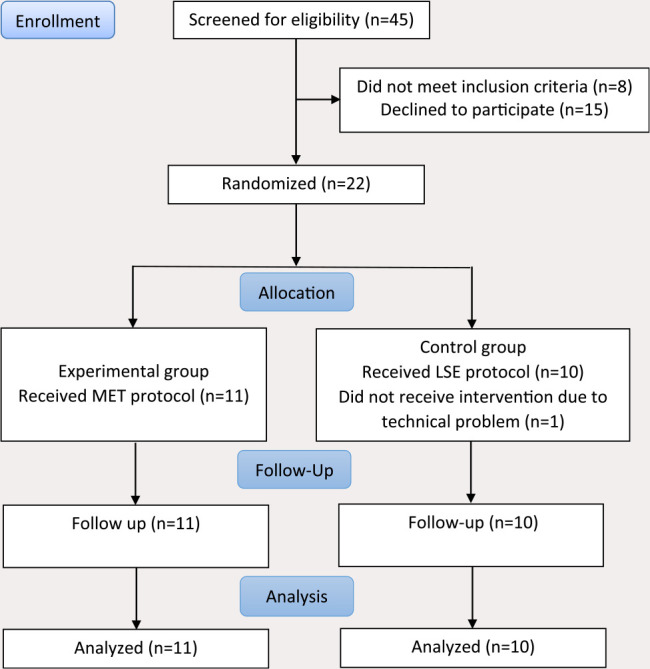
CONSORT flow chart of participant recruitment.

As shown in Fig. 1, 45 patients with chronic LBP were eligible but 37 subjects met the inclusion criteria. Twenty-two patients agreed to participate and were randomly allocated into two groups. Fifteen patients denied joining the study due to the considerably lengthy period of measurement and treatment sessions. The dataset of one subject was excluded due to the technical error during the data collection process. The characteristics of the subjects are shown in Table 1. All demographics data of both groups were normally distributed except the distribution of gender and pain location.

**Table 1.** Characteristics of subjects.

Characteristics	MET group (n=11)	LSE group (n=10)	p-value^a^
Age (years)	28.82 ± 9.26	27.70 ± 4.80	0.357
Height (cm)	160.47 ± 7.04	162.41 ± 5.98	0.508
Weight (kg)	59.00 ± 11.00	58.69 ± 12.00	0.950
BMI (kg/m${}^{2})$	23.00 ± 4.36	22.29 ± 4.01	0.745
Duration of symptom (months)	14.27 ± 16.99	11.60 ± 7.27	0.108
Gender (F/M) (n)	8/3	8/2	
Pain location	Number (%)	Number (%)	
Right lumbar	3 (27.3%)	7 (70%)	—
Left lumbar	8 (72.7%)	3 (30%)	—

*Notes*: ${}^{\text{a}}p$-value from the independent sample t-test.

The values show as $\bar{x}$ ± SD or number (n).

**Fig. 2.**
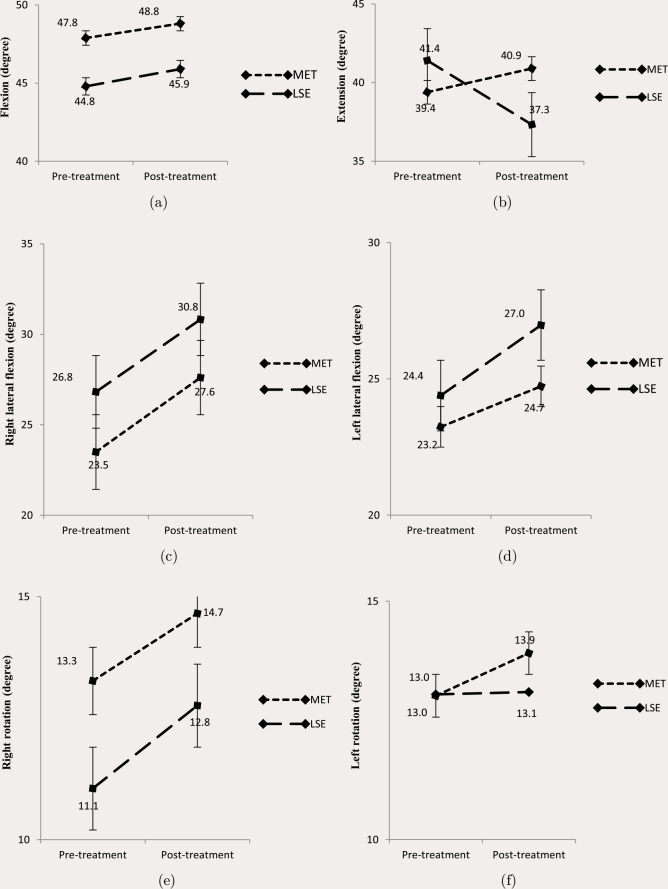
Active ROM all motions for the MET and the LSE groups.

**Fig. 3.**
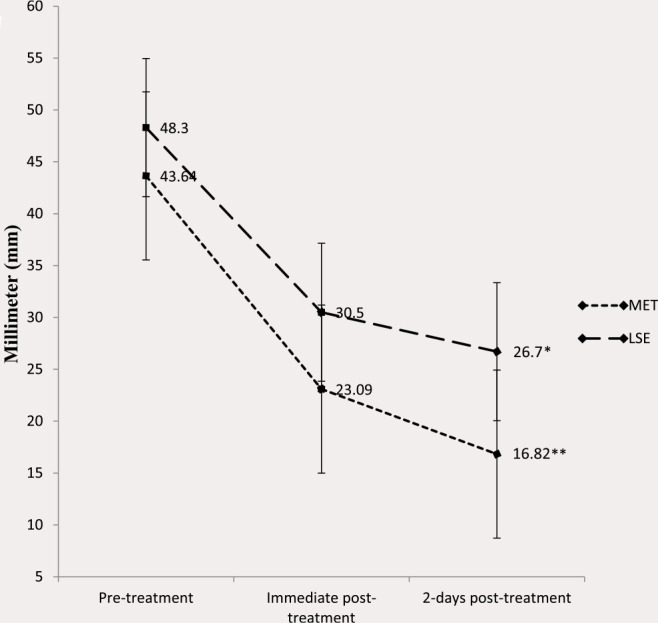
Visual analogue scale scores for the MET and the LSE groups. *Notes*: *significant difference between pre-treatment with 2-day post-treatment *p*=0.006. **significant difference between pre-treatment with 2-day post-treatment *p*<0.001.

**Fig. 4.**
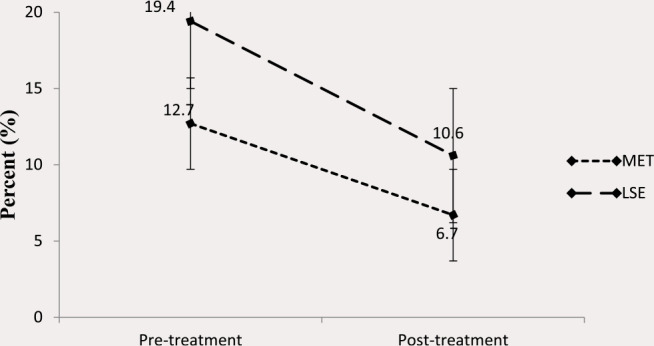
Disability scores for the MET and the LSE groups.

Table 2 shows the active ROM of lumbar spine at pre- and post-treatment, pain intensities at pre-, post-, and 2-days follow up, and the disability scores at pre and 2-day follow up of both groups. The average changes of VAS at immediately and 2-day post-treatments were 20.5 and 26.8 mm for the MET group and 17.8 and 21.6 mm for the LSE group. For ODQ, the average changes of disability scores at 2-day post-treatment were 6.0 and 8.8 for MET and LSE, respectively. The results also graphically presented in Figs. 2–4.

**Table 2.** Outcomes for MET and LSE group.

		MET		LSE			
Outcome	Evaluation	Mean score	Mean change from baseline	Mean score	Mean change from baseline	Mean difference between groups in change from baseline^a^	P value
AROM Flexion	Baseline	47.8		44.7			
		(20.65 to 76.39)		(13.66 to 82.16)			
	Immediately after	48.8	9.21	45.8	1.11	−1.88	0.95
		(20.55 to 70.49)	(−10.93 to 20.15)	(7.44 to 77.97)	(−6.22 to 9.32)	(−6.38 to 6.00)	
	2-days						
AROM Extension	Baseline	39.3		41.3			
		(11.39 to 46.67)		(33.71 to 54.25)			
	Immediately after	40.8	1.50	37.3	−4.00	5.51	0.119
		(23.31 to 61.14)	(−21.10 to 11.92)	22.23 to 47.86	(−14.74 to 4.08)	(−1.55 to 12.58)	
	2-days						
AROM Right Lateral Flexion	Baseline	23.4		26.8			
		(18.16 to 31.49)		(17.98 to 39.46)			
	Immediately after	27.6	4.11	30.8	4.01	0.10	0.954
		(21.99 to 36.61)	(−1.02 to 9.39)	(23.32 to 38.73)	(−4.36 to 12.99)	(−3.73 to 3.95)	
	2-days						
AROM Left Lateral Flexion	Baseline	23.2		24.3			
		(14.52 to 37.45)		(17.90 to 49.22)			
	Immediately after	24.7	1.48	26.9	2.58	−1.10	0.601
		(13.82 to 35.29)	(−3.69 to 6.78)	(10.83 to 47.86)	(−7.07 to 10.03)	(−5.43 to 3.23)	
	2-days						
AROM Right Rotation	Baseline	13.2		11.0			
		(6.23 to 32.23)		(6.55 to 17.49)			
	Immediately after	14.6	1.38	12.7	1.70	−0.31	0.897
		(6.32 to 26.19)	(−12.09 to 12.95)	(6.72 to 20.43)	(−2.11 to 7.08)	(−5.35 to 4.72)	
	2-days						
AROM Left Rotation	Baseline	13.0		13.0			
		(6.49 to 22.35)		(5.34 to 21.04)			
	Immediately after	13.9	0.60	13.0	0.46	0.56	0.818
		(3.77 to 30.77)	(−8.84 to 15.45)	(6.47 to 22.02)	(−4.95 to 6.26)	(−4.47 to 5.59)	
	2-days			—			
VAS scores	Baseline	43.6		48.3			
		(21 to 67)		(21 to 78)			
	Immediately after	23.0	−20.55	30.5	−17.80	−2.74	0.7.92
		(3 to 62)	(−67 to 20)	(8 to 71)	(−49 to 20)	(−24.24 to 18.75)	
	2-days	16.8	−26.82	26.7	−16.80	−10.01	0.287
		(5 to 62)	(−64 to 9)	(5 to 55)	(−42 to 17)	(−29.17 to 9.13)	
ODQ scores	Baseline	12.7		19.4			
		(6 to 30)		(6 to 60)			
	Immediately after						
	2-days	6.7	−6.0	10.6	−8.80	2.80	0.329
		(2 to 16)	(−16 to 0)	(2 to 34)	(−26 to 2)	(−3.05 to 8.65)	

*Note*:^a^ Mean differences are adjusted for baseline scores of outcome variable.

There were no significant differences of all kinematic variables (flexion, extension, right lateral flexion, left lateral flexion, right rotation, and left rotation), VAS, and ODQ scores for both MET and LSE groups at the baseline. No significant interaction effects of all kinematic variables, pain intensity, and disability level were found. The main effect analysis showed significant differences between times for right lateral flexion, VAS, and ODQ scores (p<0.001). The post-hoc analysis of VAS showed significant difference between pre-treatment with 2-day post-treatment, with pvalue=0.006 for MET group and <0.001 for LSE group.

## Discussion

This study was the first one to compare the immediate effects of the MET and the LSE in patients with chronic LBP with suspected facet joint origin. There was a trend of an increased active ROM for all directions after treatments in both groups except the extension in LSE group. No significant differences between groups were presented and the effect sizes of all movements were minimal, except for the right lateral flexion which had a medium effect size.^33^ These results might be influenced by the reflexive relaxation on spasm muscle, as most of the subjects had pain on left, especially the MET group. However, the change was not clinically meaningful according to a previous study.^30^ This finding was similar to a previous report which studied the MET effects on lumbar movements in which significant increases (5–10.5°) of ROM were observed in all directions.^34^ However, there were no significant differences of movements gained between two groups who were treated with the MET and myofascial release techniques.^34^

The results of treatment on pain were not different between two groups, and the effect size was also minimal. However, there was a significant main effect of time. The decrease in pain at immediate and two days after treatments was found when the data of both groups were combined. This implied that active exercise treatments either MET or LSE had an effect on pain in patients with chronic LBP. This result was in accordance with a previous study in which similar effects comparing MET with ultrasound were observed.^35^ However, those results should be implemented with care because they also applied the combination of treatments including moist heat, transcranial electrical nerve stimulation, and conventional exercises in both groups. These might result in the cumulative effects of several interventions.

In addition, the altered pain intensity in this study was considerably an actual change, when considering the reported minimal important change of more than 15 points or 30% improvements from the baseline interventions.^36^ The mean changes of the VAS score for both groups in this study were 19.24 points at immediately after treatments, and 24.34 points at 2-day follow-up. The decreased pain levels reflected the effectiveness of both treatments in participants with moderate pain level.

The possible explanation is the light contraction forces of both techniques which could enhance hypoalgesia in chronic pain condition both central and peripheral mechanisms.^18,20,22,37^ The rhythmic muscle contractions and stimulation of joint mechanoreceptors could affect the central mediated pathways through the stimulation of low-threshold mechanoreceptors. This would excite the neurons in the dorsal horn resulting in gating effects i.e., modulating the pain.^18^ The descending inhibition from the higher centers of central nervous system is also hypothesized.^18^ For peripheral mechanism, the light muscle contraction might stimulate fluid flow rates including blood and lymph, as well as stretch the connective tissues. The reduced pain might be associated with the change in stretch tolerance with decreasing of the muscle spindles sensitivity, and ultimately reduce pain sensitivity of both the efferent and afferent nerves.^20,22^ MET was proposed to not only break the pain or spasm cycle by inhibiting alpha motor neuron activity via a stretch reflex, but also to inhibit Ia afferent nerves via post-activation depression.^22^

For disability, while considering the main effect of time, there were statistically different disability scores between times. However, from pre-treatment to 2-day follow-up, the mean change scores were 6.0 and 8.8 for the MET group and LSE group, respectively. These scores did not meet the clinically important change of 15 points in ODI score,^38^ so a single session of both MET and LSE could not improve ODQ in patients with chronic LBP. A previous study suggested that the MET when combined with other treatments might be more effective on disability after multiple treatment sessions.^14^ Moreover, a systematic review which included 12 trials showed a low quality of evidence of the MET as the addition or comparison to other treatments on pain and disability.^39^ These findings of pain and disability levels are needed to be clarified in further studies by examining the effects of long-term and repeated MET and LSE treatment programs.

Another concern of this study is about the diagnostic tests to identify the patients with suspected pain from facet joint origin. Clinicians should be noted that the clinical criteria used in this study were based on the results of a Delphi study to gather the consensus of expert opinions.^5^ Out of the 12 criteria listed, two important indicators of “positive response to intra-articular facet joint injection” and “pain relieved by fluoroscopically guided double-anesthetic blocks of the medial branch of the dorsal ramus supplying the lumbar facet joint” were also omitted due to the nature of physical examination in physical therapy practice. Therefore, the validity of facet joint diagnosis was not confirmed.

## Conclusion

In conclusion, the different effects between MET and LSE were not found in this pilot study though attempting to include only patients with suspected pain of facet joint origin. Although this study showed statistically a significant increase of the active side-bending ROM to the painful side, as well as the decreases of the pain and disability levels, the results should be interpreted with care. The major limitation of considerably small sample size might lead to the insignificant different results. The study also monitored only immediate effect which does not reflect the usual treatment program. Further studies should be focused on long-term treatments and the evaluation with a larger sample size.

## Conflict of Interest

The authors declare that they have no competing interests.

## Funding/Support

No funding was received.

## Author Contributions

WW designed the study, evaluated the patients, analyze data and wrote the paper. MV proved the study design, treated the patients and edited the paper. SB, KM and RA proved the study design, assisted the patient recruitment, data collection, read and approved the paper.

## References

[1] Manchikanti L, Hirsch JA, Pampati V Chronic low back pain of zygapophyseal (facet) joint origin: Is there a difference based on involvement of single or multiple spinal regions? Pain Physician 2003;6:399–405. 16871288

[2] Manchikanti L, Pampati V, Fellows B, Ghafoor A The inability of the clinical picture to characterize pain from facet joints. Pain Phys 2000;3:158–66. 16906195

[3] Kalichman L, Li L, Kim DH, Guermazi A, Berkin V, O’Donnell CJ, Hoofmann U, Cole R, Hunter DJ Facet joint osteoarthritis and low back pain in the community-based population. Spine 2008;33:2560–5. 18923337 10.1097/BRS.0b013e318184ef95 PMC3021980

[4] Hestbaek L, Kongsted A, Jensen TS, Leboeuf-Yde C The clinical aspects of the acute facet syndrome: Results from a structured discussion among European chiropractors. Chiropr Osteopat 2009;17:2. 19196454 10.1186/1746-1340-17-2 PMC2642848

[5] Wilde VE, Ford JJ, McMeeken JM Indicators of lumbar zygapophyseal joint pain: Survey of an expert panel with the Delphi technique. Phys Ther 2007;87:1348–61. 17684091 10.2522/ptj.20060329

[6] Steiner C Osteopathic manipulative treatment – what does it really do? J Am Osteopath Assoc 1994;94:85–7. 8080513

[7] Peh WCG Image-guided facet joint injection. Biomed Imaging Interv J 2011;7:e4. 21655113 10.2349/biij.7.1.e4 PMC3107686

[8] Vismara L, Cimolin V, Menegoni F, Zaina F, Galli M, Negrini S, Villa V, Capodaglio P. Osteopathic manipulative treatment in obese patients with chronic low back pain: A pilot study. Man Ther 2012;17:451–5. 22658268 10.1016/j.math.2012.05.002

[9] Chaitow L Muscle Energy Techniques. 4th ed. Philadelphia, PA: Churchill Livingstone, 2013:169.

[10] Smith M, Fryer G A comparison of two muscle energy techniques for increasing flexibility of the hamstring muscle group. J Bodyw Mov Ther 2008;12:312–7. 19083689 10.1016/j.jbmt.2008.06.011

[11] Shadmehra A, Hadiana MR, Naiemib SS, Jalaiea S Hamstring flexibility in young women following passive stretch and muscle energy technique. J Back Musculoskelet Rehabil 2009;22:143–8. 20023343 10.3233/BMR-2009-0227

[12] Day JM, Nitz AJ The effect of muscle energy techniques on disability and pain scores in individuals with low back pain. J Sport Rehabil 2012;21:194–8. 22622384 10.1123/jsr.21.2.194

[13] Wilson E, Payton O, Donegan-Shoaf L, Dec K Muscle energy technique in patients with acute low back pain: A pilot clinical trial. J Orthop Sports Phys Ther 2003;33:502–12. 14524509 10.2519/jospt.2003.33.9.502

[14] Selkow NM, Grindstaff TL, Cross KM, Pugh K, Hertel J, Saliba S Short-term effect of muscle energy technique on pain in individuals with non-specific lumbopelvic pain: A pilot study. J Man Manip Ther. 2009;17:E14–8. 20046557 10.1179/jmt.2009.17.1.14E PMC2704351

[15] Kanchan R, Nitesh B, Savita Comparative analysis on the efficacy of G.D. Maitland’s concept of mobilization & muscle energy technique in treating sacroiliac joint dysfunction. Indian J Physiother Occup Ther 2009;3(2):18–21.

[16] Supreet B, Mithilesh K, Preet SP, Jagmohan S A study on the efficacy of muscle energy technique as compared to conventional therapy in chronic low back pain due to sacroiliac joint dysfunction. Indian J Physiother Occup Ther. 2012;6(1):200–3.

[17] Mitchell JFL, Mitchell PKG The MET manual. East Lansing, Michigan: MET Press; 1995.

[18] Fryer G, Fossum C Therapeutic mechanisms underlying muscle energy approaches In: Fernandez-de-las-Penas C, Arendt-Nielsen L, Gerwin RD, editors. Tension-type and cervicogenic headache: Pathophysiology, diagnosis, and management. Sudbury, Massachusetts: Jones and Barrlett Publishers, 2010: 221–229.

[19] Fryer G Research-informed MET concepts and practice In: Franke H, editor. MET technique: History-model-research (monograph). Ammersestr: Jolandos: 2009: 57–62.

[20] Fryer G MET technique: Research and efficacy In: Chaitow L, editor. Muscle Energy Techniques. 3rd ed. Edinburgh: Churchill Livingstone, 2006:109–132.

[21] Fryer G Muscle energy technique: An evidence-informed approach. Int J Osteopath Med 2011;14:3–9.

[22] Wilson E MET in the physical therapy setting In: Chaitow L, editor. Muscle Energy Techniques. Edinburgh: Churchill Livingstone; 2006:273–297.

[23] Fryer G Muscle energy concepts-a need for change. J Osteopath Med 2000;3:54–9.

[24] Goldby L, Moore A, Doust J, Trew M A randomized controlled trial investigating the efficacy of musculoskeletal therapy on chronic back disorder. Spine 2006;31:1083–93. 16648741 10.1097/01.brs.0000216464.37504.64

[25] May S, Johnson R Stabilisation exercises for low back pain: A systematic review. Physiotherapy 2008;94:179–89.

[26] Vongsirinavarat M, Wahyuddin W, Adisaiphaopan R The agreement of clinical examination for low back pain with facet joint origin. Hong Kong Physiother J 2018;38:1–7. 30930584 10.1142/S1013702518500105 PMC6405352

[27] Schache AG, Blanch P, Rath D, Wrigley T, Bennell K Three-dimensional angular kinematics of the lumbar spine and pelvis during running. Hum Mov Sci 2002;21:273–93. 12167303 10.1016/s0167-9457(02)00080-5

[28] Tojima M, Ogata N, Yozu A, Sumitani M, Haga N Novel 3-dimensional motion analysis method for measuring the lumbar spine range of motion. Spine 2013;38:E1327–33. 23797505 10.1097/BRS.0b013e3182a0dbc5

[29] Huskisson EC Measurement of pain. Lancet 1974;2:1127–31. 4139420 10.1016/s0140-6736(74)90884-8

[30] Sakulsriprasert P, Vachalathiti R, Vongsirinavarat M, Kantasorn J Cross-cultural adaptation of modified Oswestry low back pain disability questionnaire to Thai and its reliability. J Med Assoc Thai 2006;89:1694–701. 17128846

[31] De Stefano LA Greenman’s Principles of Manual Medicine. 4th ed. Baltimore, MD: Lippincott Williams & Wilkins, 2011:301–302.

[32] Richardson C The time to move forward In: Richardson C, Hodges PW, Hides J, editors. Therapeutic Exercise for Lumbopelvic Stabilization: A Motor Control Approach for the Treatment and Prevention of Low Back Pain. 2nd ed. Sydney, Churchill Livingstone, 2004:3–7.

[33] Cohen J Statistical Power Analysis for the Behavioral Sciences. 2nd ed. New York: Lawrence Erbaum Associates; 1988.

[34] Ellythy MA Efficacy of muscle energy technique versus myofascial release on function outcome measures in patients with chronic low back pain. Bull Fac Phys Ther Cairo Univ 2012;17:51–7.

[35] Bindra S, Kumar M, Singh P, Singh J A study on the efficacy of muscle energy technique as compared to conventional therapy in chronic low back pain due to sacroiliac joint dysfunction. Ind J Physiother Occup Ther 2012;6:200–3.

[36] Ostelo RW, Deyo RA, Stratford P, Waddell G, Croft P, Korff MV, Bouter LM, de Vet HC Interpreting change scores for pain and functional status in low back pain. Spine 2008;33:90–4. 10.1097/BRS.0b013e31815e3a10 18165753

[37] Hoeger Bement MK, Dicapo J, Rasiarmos R, Hunter SK Dose response of isometric contractions on pain perception in healthy adults. Med Sci Sports Exer 2008;40:1880–89. 10.1249/MSS.0b013e31817eeecc 18845975

[38] Fairbank JCT Why are there different versions of the Oswetry Disability Index? A review. J Neuosurg Spine 2014;20:83–6. 10.3171/2013.9.SPINE13344 24206036

[39] Franke H, Fryer G, Ostelo RW, Kamper SJ Muscle energy technique for non-specific low-back pain. A Cochrane systematic review. Int J Osteopath Med 2016;20:41–52. 10.1002/14651858.CD009852.pub2 PMC10945353 25723574

